# Weak Polygyny in California Sea Lions and the Potential for Alternative Mating Tactics

**DOI:** 10.1371/journal.pone.0033654

**Published:** 2012-03-14

**Authors:** Ramona Flatz, Manuela González-Suárez, Julie K. Young, Claudia J. Hernández-Camacho, Aaron J. Immel, Leah R. Gerber

**Affiliations:** School of Life Sciences, Arizona State University, Tempe, Arizona, United States of America; University of Western Ontario, Canada

## Abstract

Female aggregation and male territoriality are considered to be hallmarks of polygynous mating systems. The development of genetic parentage assignment has called into question the accuracy of behavioral traits in predicting true mating systems. In this study we use 14 microsatellite markers to explore the mating system of one of the most behaviorally polygynous species, the California sea lion (*Zalophus californianus*). We sampled a total of 158 female-pup pairs and 99 territorial males across two breeding rookeries (San Jorge and Los Islotes) in the Gulf of California, Mexico. Fathers could be identified for 30% of pups sampled at San Jorge across three breeding seasons and 15% of sampled pups at Los Islotes across two breeding seasons. Analysis of paternal relatedness between the pups for which no fathers were identified (sampled over four breeding seasons at San Jorge and two at Los Islotes) revealed that few pups were likely to share a father. Thirty-one percent of the sampled males on San Jorge and 15% of the sampled males on Los Islotes were assigned at least one paternity. With one exception, no male was identified as the father of more than two pups. Furthermore, at Los Islotes rookery there were significantly fewer pups assigned paternity than expected given the pool of sampled males (p<0.0001). Overall, we found considerably lower variation in male reproductive success than expected in a species that exhibits behavior associated with strongly polygynous mating. Low variation in male reproductive success may result from heightened mobility among receptive females in the Gulf of California, which reduces the ability of males to monopolize groups of females. Our results raise important questions regarding the adaptive role of territoriality and the potential for alternative mating tactics in this species.

## Introduction

Polygyny, where one male mates with multiple females within a breeding season, is common among species where females bare the burden of gestation and care of offspring. Unconstrained by parental duties, a male's reproductive success is only limited by the number of females he can fertilize [1], [2]. Aggregations of sexually receptive females allow males to monopolize groups of females [3] and it is the ensuing competition among males for access to potential mates that determines the degree of polygyny, or variation in male reproductive success [1]–[3]. It follows that male behavior during the breeding season has been used to infer the mating system and degree of polygyny within a population. However, the use of molecular genetic techniques in the study of mating systems has highlighted discrepancies between behavioral and genetic mating systems [4]. Indeed, paternity analysis in polygynous mating systems has revealed that males may not always be as successful at monopolizing access to breeding females as assumed from behavioral observations and theory [5]–[7]. In this study we use genetic tools to explore the mating system of a behaviorally polygynous species, the California sea lion (*Zalophus californianus*) in the Gulf of California, Mexico.

Pinnipeds (seals and sea lions) exhibit a variety of mating strategies which range from monogamy (e.g., largha seal, *Phoca largha*) to strong polygyny (e.g., elephant seals, genus *Mirounga*, Steller sea lion, *Eumetopias jubatus*) [8], making them ideal for the study of mating systems. Polygyny is facilitated by the aggregation of females during the breeding season as they haul out to give birth and nurse their pups [8]–[15]. Males defend spatially stable territories within a rookery and females either 1) mate with the male whose territory they primarily occupy (i.e., resource defense polygyny); or 2) freely move between male territories and select mates based on territorial displays, creating a lek-like system [8], [10].

California sea lions breed from May to August on islands along the Pacific coast of the continental U.S. and the Baja peninsula, as well as the Gulf of California, Mexico [8], [16], [17]. In May and June, female California sea lions haul out and give birth to a single pup [18] which they nurse for about five days before leaving the breeding colony, or rookery, on short foraging trips lasting approximately three days [19]. During the breeding season, pups have very limited mobility and are dependent on their mother's milk for survival. Thus, females routinely return to their pupping site to nurse [8]. Adult males, which are nearly three times larger than females [20], defend territories occupied by females and pups [9], [21]–[23]. Overall, the species' high sexual dimorphism, and male territorial behavior are suggestive of moderate to strong polygyny [8], [9], [15], [24], [25].

Sea lion populations in the Gulf of California (Gulf) represent an interesting study system for several reasons. First, in the Gulf adult males actively defend territories with male tenure averaging around two weeks [26] but extending more than six weeks in some cases [19]. Although males defend territories, direct observation of copulations is rare at our study sites [19] compared to other California sea lion rookeries [14], [19]. Second, female sea lions in the Gulf spend a considerable portion of their time at the rookeries in the water. This behavior is presumably a thermoregulatory response to exposure to the extremely high temperatures (up to 45°C) in the Gulf [19], [27]. In the water, females have greater mobility which likely limits the ability of males to monopolize them and may reduce the degree of polygyny [19]. Finally, there is a prolonged period between parturition and estrous, which extends for >30 days on average for females in the Gulf [18], as compared to 21 days for females off the California coast [14]. Thus, females within the Gulf become sexually receptive at a time when they are increasingly mobile and routinely leave the island on foraging trips when maturing pups are able to survive for several days between nursing bouts.

The extent to which these characteristics of sea lion breeding biology and behavior in the Gulf populations affect the mating dynamics is unclear, but it raises interesting questions about the mating system in these populations. In particular, it has been suggested that sea lions may exhibit a lek-like mating system where males defend territories and females move freely between territories to select mates [18], [19], [21]. This is in contrast to classic resource defense polygyny where sexually receptive females mate with the male whose territory they occupy. Alternatively, Odell [14] theorized that female movement in sea lions may facilitate alternative mating tactics, reducing male reproductive skew and, perhaps, even giving non-territorial males an advantage. The lack of genetic studies to validate these competing hypotheses has left the debate over the nature of California sea lion mating systems largely unresolved. In response to this need for research on the genetic mating system of California sea lions, we used genotype data from 14 polymorphic microsatellite loci to evaluate the type of polygyny (resource defense vs. a lek-like system), the degree of male reproductive skew, and the potential for alternative mating tactics present at three breeding sites on two sea lion rookeries in the Gulf.

## Methods

### Ethics statement

All procedures were approved by the Arizona State University Animal Care and Use Committee (07-918R). Data collection was authorized by the Secretaría de Medio Ambiente y Recursos Naturales (SEMARNAT, Oficio num/SGPA/DGVS/05325/05, 03269/06, 02709/07, and 03018/08). Samples were imported from Mexico under permits by SEMARNAT (Autorización no. 20215, 21572, 23010, and 24575) and the US National Marine Fisheries Service (no. 782-1694-02).

### Study sites

**Figure 1:**
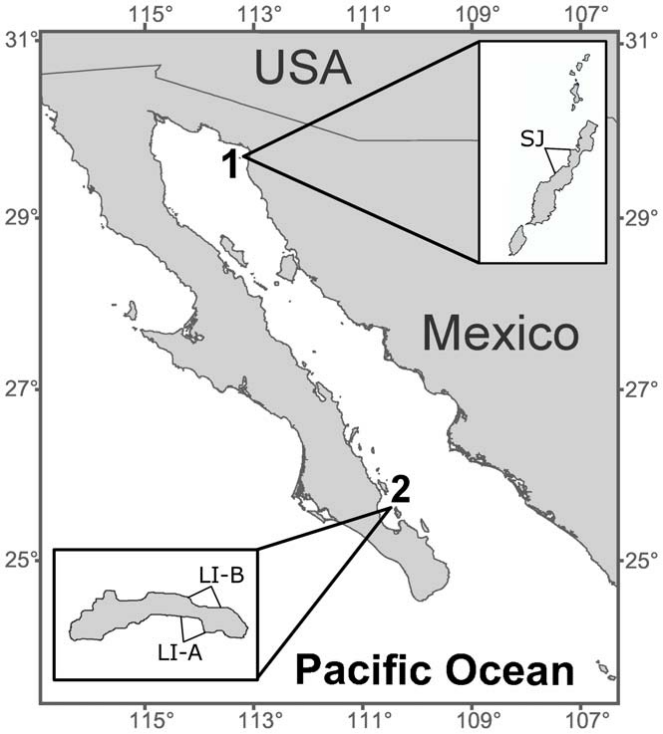
Map of the study sites at two sea lion rookeries in the Gulf of California. Insets indicate sampled study areas within each rookery. 1) San Jorge Island measures approximately 2.0 km between the northern islet and southern tip and 0.2 km at the widest point. Here, the marked area includes the study site and sampled adjacent areas. 2) Los Islotes Island is approximately 0.5 km long and 0.1 km at its widest point.

For this study we selected two sea lion rookeries in the Gulf which had been the subject of behavioral research since 2004 [28]–[30], San Jorge and Los Islotes Islands (Fig. 1). These rookeries support sea lion populations of approximately 4,000 and 500 individuals, respectively [17]. Field research took place from 2005 to 2008 at San Jorge and from 2006 to 2008 at Los Islotes. Each field season consisted of 2–3 trips (each lasting 5–10 days) in June, July, and August. During each trip we collected behavioral data and tissue samples from two study sites on Los Islotes and one on San Jorge (Fig. 1). Study sites were representative of typical sea lion habitat, characterized by concave stretches of the coastline yielding a relatively high concentration of females and pups [31].

At San Jorge, females, pups, and territorial males occupy virtually the entire coastline. Our study site included a section of the coast with a discrete cobble cove and up to eight male territories where we conducted behavioral observations and captured pups. Because we observed some females and pups moving in and out of the study site along the coastline, we also collected tissue samples from individuals in neighboring areas extending approximately 80 m along the coast on either side of the cove. In 2008, we collected samples from males at seven additional areas across the rookery to investigate relatedness patterns. At Los Islotes, females and pups haul out primarily along three distinct areas of the coast due to the near vertical shoreline along other parts of the island. Of the three breeding areas on Los Islotes, the two areas with the highest concentration of females and pups were sampled in our study (Fig. 1).

### Tissue collection

Tissue samples were obtained from pups by clipping a small (<1 g) sample of skin from the tip of a hind toe. Toe clips were placed in a 2 ml screw top tube containing 95% ethanol labeled with the date and a unique pup code. Scalpels and tweezers were cleaned between each biopsy with 100% ethanol and a clean kimwipe®. Individuals were identified with a unique haircut, which remained visible until late September of each year.

Tissue samples from adults were obtained using a crossbow and bolts fitted with custom made biopsy tips (Quality Manufacturing, Inc.) to which fishing line was attached for sample retrieval, (see [32]). To obtain samples of female-pup pairs for paternity analysis, we only took biopsies from females who were nursing a marked pup (from which a tissue sample had already been collected). California sea lions have been found to occasionally nurse non-filial pups [33], however nursing behavior is still the most reliable indicator of true female-pup pairs and non-filial pairs can be easily identified by mismatches between female and pup genotypes; genotype mismatches are described in detail in the section on *paternity assignment*. Territorial males were identified based on scar patterns [21], allowing observers to track individual males between and throughout observation trips. At each island we obtained biopsies from territorial males within each study site, and on San Jorge we sampled additional territorial males adjacent to the site as described above.

### DNA extraction, amplification, and analysis

DNA was extracted from tissue samples using a simple salting out procedure [34] and stored at −20°C. Once extracted, DNA was amplified at 14 microsatellite loci developed for California sea lions or closely related species (Table 1) in four multiplex polymerase chain reactions (PCR) using the Qiagen multiplex kit (Qiagen Inc.). We modified the microsatellite protocol outlined in the 2007 Qiagen Muliplex PCR Handbook so that 0.10 µM of each primer was used per reaction, and total reaction volume was 7 µl. To monitor for contamination, negative controls were included in each manipulation. Fragment analysis was conducted on an ABI 3730 DNA Analyzer (Applied Biosystems, Inc.) and peaks were called by hand using the program GeneMapper v4.0. To ensure that each individual was only represented once in the database, the program GIMLET [35] was used to identify samples with identical genotypes. Samples with identical genotypes were considered to be from the same individual.

**Table 1:** The number of observed alleles (N_a_), expected heterozygosity (H_E_), and non-exclusion probability for paternity assignment for 14 microsatellite loci.

	San Jorge			Los Islotes		
Locus	N_a_	H_E_	Non-exclusion probability	N_a_	H_E_	Non-exclusion probability
Pv09 [50], [51]	6	0.482	0.7113	7	0.496	0.7122
Pv11 [50], [51]	4	0.488	0.7209	5	0.544	0.6656
ZcCgDh4.7 [51]	3	0.460	0.7902	3	0.461	0.7727
ZcCgDh5.16 [51]	9	0.782	0.4293	6	0.693	0.5524
ZcCgDh1.8 [51]	6	0.663	0.5925	6	0.666	0.5918
ZcCgDh48 [51]	4	0.619	0.6684	5	0.536	0.7187
ZcCgDh5.8 [51]	10	0.756	0.4432	11	0.815	0.3595
OrrFCB24 [52], [53]	11	0.850	0.2994	11	0.840	0.3097
Pvc29 [53], [54]	16	0.842	0.3126	16	0.880	0.2479
ZcCgDh3.6 [51]	6	0.630	0.5841	7	0.551	0.6467
Hg6.1 [51], [53], [55]	5	0.635	0.6530	9	0.687	0.5655
Hg8.10 [53], [55]	5	0.541	0.7172	7	0.683	0.5668
13HDZ462 [56]	4	0.518	0.7592	4	0.634	0.6740
71HDZ5A [56]	9	0.632	0.5816	9	0.737	0.4286
*Summary*	*7.00*	*0.6355*	*0.0003*	*7.57*	*0.6611*	*0.0001*

Summary values include the average number of alleles and heterozygosity per locus, and the combined non-exclusion probability across all loci.

### Paternity assignment

Because potential fathers were sampled a year prior to the year their offspring were sampled (i.e. the year of conception), we did not include female-pup pairs from the first field season for paternity assignment. We used the program cervus [36] to identify mismatches between female and pup genotypes (i.e., loci at which no alleles were shared). When mismatches were identified, we repeated extraction and/or PCR for the samples in question. If mismatches persisted, the female-pup pair was not used in paternity analyses. Therefore, only confirmed filial female-pup pairs were analyzed. Because the number of potential fathers available at each island was unknown, we assumed that 50% of the candidate males were sampled. This is the lowest fraction of candidate males advised by cervus; exclusion probabilities below this threshold may be inaccurate. We included all sampled adult males in the list of candidate fathers, meaning that cervus was allowed to consider all possible female-pup-male trios for each rookery, even if the male was not sampled the year of conception.

Genotyping error or the presence of null alleles can affect the results of paternity analysis (Hoffman et al. 1999). Repeated genotyping of 50 individuals at all 14 loci resulted in one, single-locus genotype mismatch, giving a locus error rate of 0.0014. Even a low genotyping error rate can have a large impact on the outcome of genetic paternity assignment [37], [38], so to further reduce errors in paternity assignment due to genotyping error, PCR and genotyping was repeated for all samples from female-pup-male trios that mismatched at two or fewer loci. To identify loci containing null alleles, the program cervus was used to test for deviations from Hardy-Weinburg equilibrium. Paternity assignments were only considered valid if a single candidate male matched a female-pup pair at all 14 loci and with 95% confidence (i.e., ≥95% chance of the male being the father).

We examined the probability that females are more likely to sire pups with males holding territories within each site than with males holding territories elsewhere on each rookery. The total number of males holding territories throughout the breeding season was estimated for both islands using census data from the entire island combined with information on male turnover within territories at our study sites. We then calculated the percentage of pups expected to be assigned paternity if females were equally likely to mate with any one of the territorial males, regardless of territory location. The expected and observed percentage of paternity assignments were compared using a binomial distribution where each pup that was assigned a father was considered a success and each pup not assigned a father was considered a failure. Paternity matches in which the father was not observed or sampled the year of conception were not included in this analysis. At San Jorge, where males holding territories directly adjacent to the study site were sampled, separate analyses were done including males from the adjacent areas and excluding them. All female-pup pairs were used in each analysis because movement of females and pups, particularly during capture sessions, made it impossible to differentiate between female-pup pairs that primarily occupied territories within the site and those that occupied territories in areas directly adjacent to the site. Data from sites A and B at Los Islotes were combined because movement of females and pups occurs between these two sites. To increase power, we combined information from all years at each site.

### Paternal relatedness

Measurements of paternal relatedness between pups were used to estimate relative male reproductive success from the subset of female-pup pairs for which no father was identified. This was done using the Microsoft Excel Macro, dadshare [39] in which paternal haplotypes were derived from the genotypes of known female-pup pairs and systematically organized into a dendrogram according to relatedness. Clusters of pup genotypes consistent with a single male were identified to determine the minimum number of fathers necessary to account for the total pool of sampled pups at each island. This results in the highest possible ratio of pups per successful male and is thus expected to overestimate variance in male reproductive success. Therefore, to obtain a more accurate estimate of male reproductive success, mean pairwise relatedness between external branches of the dendrogram was compared to similar results from Monte Carlo simulations of five scenarios representing strong polygyny with either 1, 2, 3, 4, or 5 fathers siring all offspring (a few males contributing to the sample of pups), and five scenarios representing weak polygyny with each successful male siring 1, 2, 3, 4 or 5 pups (many males contributing to the sample of pups). For this analysis, female-pup pairs at each island were combined for all years (2005–2008 on San Jorge, and 2007–2008 on Los Islotes). To evaluate the accuracy of the dadshare approach for our sample, the dataset of pups that were assigned fathers were analyzed independently and results were compared with the true ratio of pups per father as determined by paternity assignment.

## Results

### Sample collection and genotype analysis

At San Jorge, 118 female-pup pairs and 65 males were sampled. Of these, eight females were sampled twice in different years and four females were sampled in three different years, where each successive sampling included a new pup from that year. Thirty-six of the female-pup pairs were sampled in the first year (2005). These samples were used in analysis of paternal relatedness but not for paternity assignment because potential fathers (males from 2004) were not sampled. At Los Islotes, 40 female-pup pairs and 34 males were sampled. One female was sampled in both years. Each year we sampled approximately 75% of the territorial males identified in each site, except for Los Islotes rookery in 2006 when we only obtained biopsies from approximately 35% of the observed territorial males. The distribution of samples from males and female-pup pairs used in paternity analyses are shown in Table 2.

**Table 2:** Distribution of sampled individuals used in paternity analyses.

	San Jorge			Los Islotes A		Los Islotes B	
Year	Female-pup pairs	Males (site)	Males (adjacent areas)	Female-pup pairs	Males	Female-pup pairs	Males
2005	36	11	12	-	-	-	-
2006	26	12	20	-	9	-	5
2007	23	11	23	10	13	17	13
2008	33	-	-	8	-	5	-
Total	118	21	44	18	19	22	15

Males that were present in multiple years are counted once for column totals.

No evidence of null alleles was found and all loci were in Hardy-Weinberg equilibrium. Repeated genotyping indicated a very low genotyping error rate of 0.0014 per locus and even when this error rate was doubled the outcome for paternity assignment did not change. No male that matched a female-pup pair at all loci was assigned paternity with less than 95% confidence and no male that mismatched a female-pup pair at one or more loci was assigned paternity with over 80% confidence. Thus, although we only assigned paternities when males matched female-pup pairs at all loci and with 95% confidence, identical results would have been obtained with a strict exclusion approach, or a likelihood based approach with either a 95% or 80% confidence threshold.

### Paternity assignment

At San Jorge, paternity assignment included 82 female-pup pairs sampled from 2006–2008 and 65 territorial males sampled from 2005–2007. Paternities were assigned to 30% of the female-pup pairs and included 31% of the sampled males. Twenty-three percent of sampled males both sired offspring and were observed at the rookery the year of conception and these paternities accounted for 23% of the pups. Of the 21 sampled males who held territories within the sample site (excluding adjacent areas), 29% were assigned paternity to a total of 12% of the female-pup pairs. Only one of these pups was sired by a male that was not observed the year of conception.

At Los Islotes, paternity assignment included 40 female-pup pairs sampled in 2007–2008 and 34 territorial males sampled in 2006–2007. Fifteen percent of the sampled males could be assigned paternity to a total of 15% pups. When only paternities assigned to males that had been observed the year of conception were considered, these numbers were reduced to 12% of the males and 10% of the pups.

**Figure 2:**
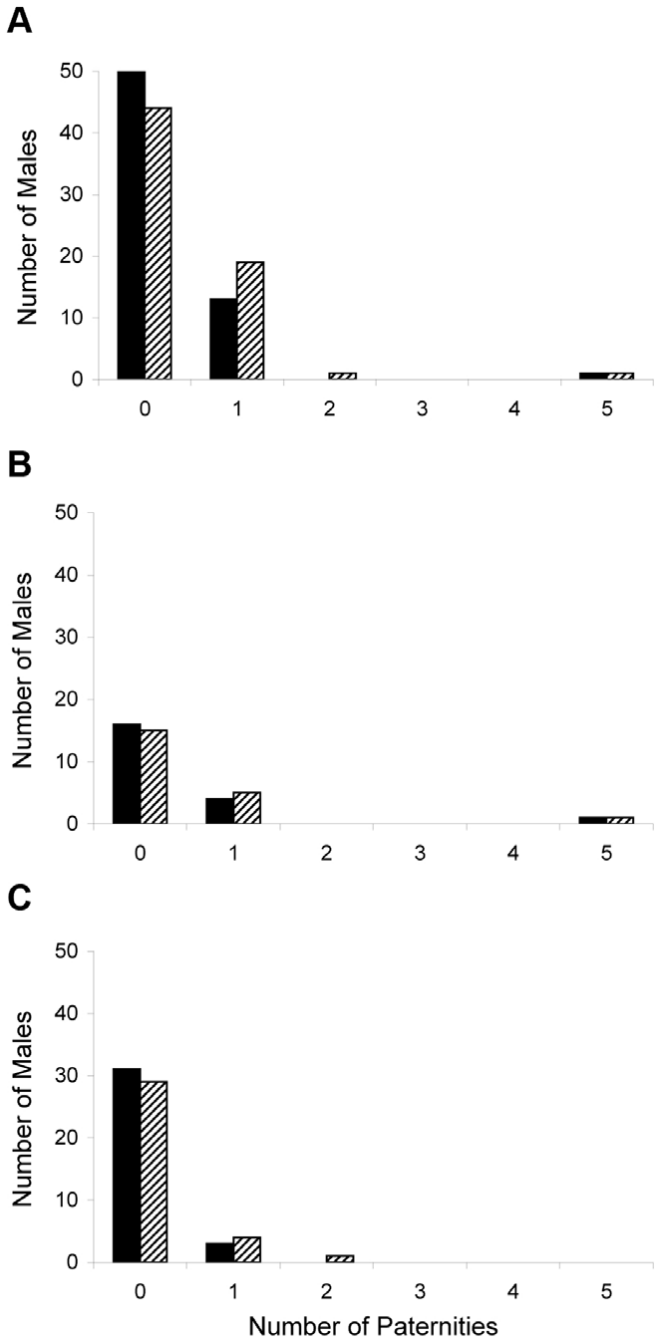
Distribution of cumulative male reproductive success. Bars represent the number of paternities assigned over a three year period to each of a) 65 territorial males on San Jorge including males both within the study site and along adjacent areas and b) 21 territorial males on San Jorge including only those males within the study site, and c) the number of paternities assigned over a two year period to each of 35 territorial males on Los Islotes. Solid bars include only those paternities where the father was identified at the rookery the year of conception, whereas hatched bars include all paternity assignments.

Both islands had similar patterns of paternity assignment with over 65% of the males not assigned any paternity. Most identified fathers were assigned paternity to a single pup (Fig. 2). Males with multiple assigned paternities had two offspring, except a single male who sired five pups born in 2007 and 2008. This male defended a territory within the study site on San Jorge.

Using census and male turnover data, we estimated 250 males held territories at San Jorge each year during the breeding season. Pups were more likely to be assigned a father from the pool of sampled males than would be expected if females were equally likely to mate with any one of the territorial males throughout the entire rookery (p = 0.0157, power: 1-β = 0.5466). This was slightly more pronounced when only those males holding territories within the site were considered and males from adjacent areas were excluded (p = 0.0024, power: 1-β = 0.6874) (Table 3). The number of years in which a male was observed at a site did not differ between males that were assigned paternities (n = 6, mean±SD = 2.0±0.7 years) and males that were not (n = 15, mean±SD = 1.8±0.5 years).

**Table 3:** Comparisons of observed and expected rates of paternity assignment.

Year	Males (t-1)	Female- pup pairs	Observed paternities	Expected paternities	p-value	1-β
**San Jorge**						
2006	23	15	2 (13.33%)	1.4 (9.20%)	0.3082	0.0394
2007	33	22	6 (27.27%)	2.9 (13.20%)	0.0390	0.3811
2008	34	33	7 (21.21%)	4.5 (13.60%)	0.1411	0.1440
2006–2008	28	70	15 (21.43%)	8.4 (12.00%)	0.0157	0.5466
**San Jorge (excluding adjacent areas)**						
2006	11	15	2 (13.33%)	0.7 (4.40%)	0.0521	0.3188
2007	12	22	3 (13.64%)	1.1 (4.80%)	0.0388	0.1364
2008	11	33	4 (12.12%)	1.5 (4.40%)	0.0280	0.3626
2006–2008	11.3	70	9 (12.86%)	3.2 (4.53%)	0.0024	0.6874
**Los Islotes**						
2007	14	27	2 (7.41%)	7.56 (28.00%)	0.0182	0.6774
2008	26	13	1 (7.69%)	6.76 (52.00%)	0.0022	0.9294
2007–2008	20	40	3 (7.50%)	7.16 (40.00%)	<0.0001	0.9994

Paternity assignment rates for San Jorge include all sampled territorial males and only those males sampled within the study site (excluding adjacent areas). At Los Islotes data from sites A and B were combined. The males for each year were actually sampled the year of conception (t−1). Expected paternities are calculated under the assumption females are equally likely to mate with any territorial male, regardless of territory location. P-values from a two-tailed binomial test and power (1−β) for p = 0.05 are reported.

At Los Islotes, we estimated a maximum of 50 males held territories throughout each breeding season. Sampled males were less likely to be assigned parentage than expected at random (p<0.0001, power: 1-β = 0.9994) (Table 3), suggesting that the estimated pool of territorial males on the island is not sufficient to account for the pups born on the island each year.

### Paternal relatedness

**Figure 3:**
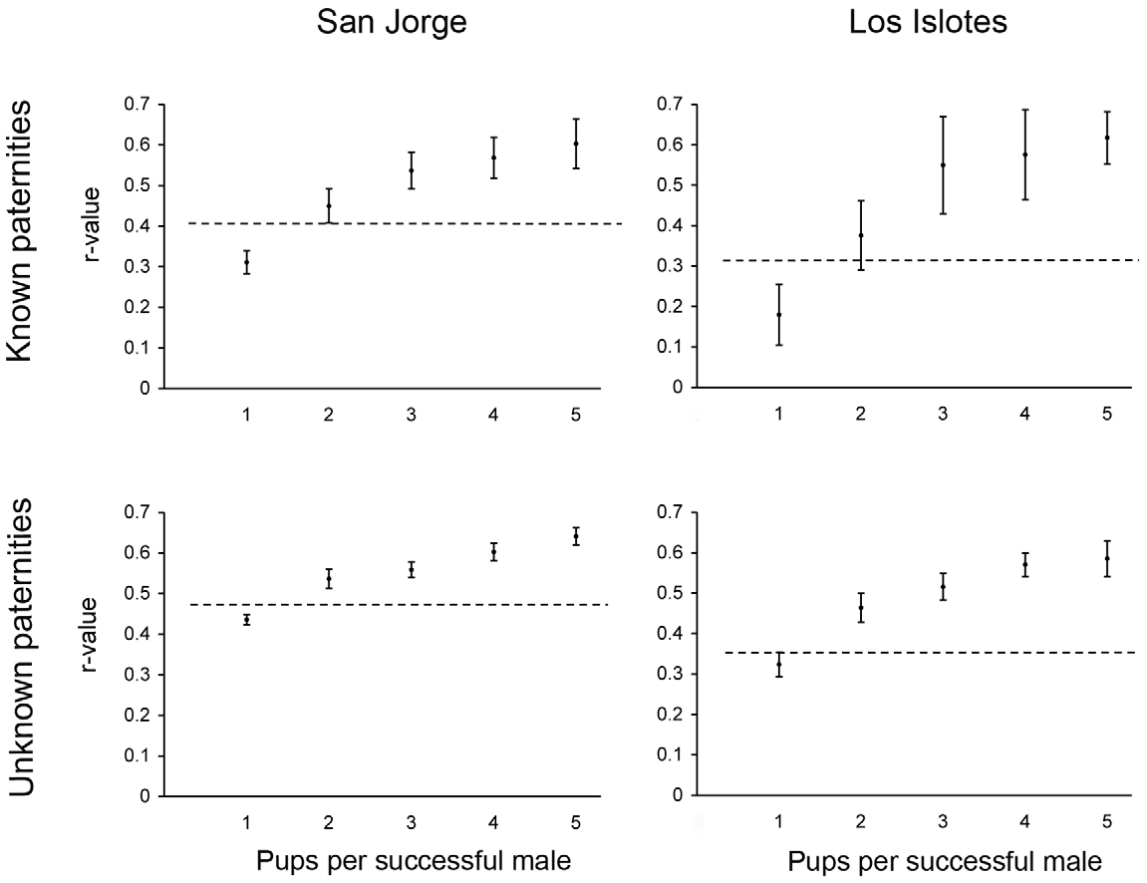
Observed paternal relatedness compared with expected values over a range of polygyny levels. Expected values of average paternal relatedness (r-values) between pups if each successful male sires 1, 2, 3, 4 or 5 pups. Error bars represent +/− one standard deviation. Horizontal lines represent the observed r-values for each pool of sampled pups. At each of the two rookeries, separate analyses were done for pups that were assigned fathers (known paternities) and pups whose fathers could not be assigned (unknown paternities). In all cases observed r-values fall between what would be expected if each successful male sired just one or two pups.

Comparisons of paternal haplotypes from pups sampled over four years at San Jorge and two years at Los Islotes using the program dadshare revealed that a minimum of 39 males was necessary to account for the 69 pups with unknown fathers at San Jorge and a minimum of 19 males was necessary to account for the 34 pups with unknown fathers at Los Islotes. It was uncommon that groups of more than two pups were compatible with a single father, and five pups on Los Islotes constituted the largest group which could have potentially shared a father. Coincidentally, this was also the largest number of paternities assigned to a single male in this study (one male at San Jorge). The actual ratio of pups per male for the sample of pups with unknown fathers is probably closer to 1∶1, as shown by comparing the observed mean values of paternal relatedness with values expected under simulations of varying numbers of pups per male (Fig. 3). These values are consistent with the paternity assignment results, where all but one male sired either one or two pups. Evaluating paternal relatedness patterns for the pool of pups with known fathers resulted in a pup per male ratio closer to 2∶1 (Fig. 3). This suggests that dadshare may tend to overestimate the level of polygyny in our data.

## Discussion

Many pinniped populations (including the California sea lion populations in this study) exhibit aggregations of breeding females and male territoriality, and, as expected given these behaviors, are often strongly polygynous [3], [10], [40]. For example, in the Antarctic fur seal (*Arctocephalus gazella*), less than 10% of successful males have been shown to share a quarter of assigned paternities [39], and a recent study of grey seals (*Halichoerus grypus*) revealed a mating system where nearly half the pups were fathered by 13% of territorial males present on the rookery [41]. Support for comparable polygyny levels in California sea lions comes from a behavioral study on copulation frequency and distribution at three rookeries (two off the California coast, and one in the Gulf of California). At all three rookeries, a small proportion of the males accounted for the majority of observed copulations suggesting a strongly polygynous system [19]. However, our results show little reproductive skew among males, where only one male was identified as the father for more than two pups across four breeding seasons at San Jorge Island and three breeding seasons at Los Islotes Island. This male was assigned five paternities during his two-year tenure at the study site.

A recent paternity study of a closely related species, the Galapagos sea lion (*Zalophus wollebaeki*), revealed weak polygyny [7] comparable to our results. These authors found that in each breeding season, most sampled males sired no pups and most successful males sired only one or two pups with no male siring over four pups [7]. The authors hypothesized that their results are explained by the extended, non-synchronous breeding season of the Galapagos sea lion [42], which limits the ability of males to monopolize females and weakens polygyny. Interestingly, our research shows a nearly identical pattern of paternity assignments for a species with a synchronous mating season, suggesting that other factors may play a role in determining the extent of male reproductive skew. Odell [14] proposed that the ability to mate aquatically and a prolonged period between parturition and estrus may allow a less polygynous system to develop via a reduced ability of males to monopolize groups of receptive females. Like California sea lions in the Gulf, Galapagos sea lions are exposed to warm temperatures and have a prolonged period between birth and estrous [43]. These patterns suggest that changes in behavior associated with thermoregulation and an extended period between birth and estrous may be the critical factors limiting the ability of males to monopolize females. Whether asynchrony in mating also plays a role in limiting monopoly of females remains to be clarified. However, our results show that asynchrony is not required to reduce the levels of polygyny in otariids (fur seals and sea lions) and that weak polygyny may be more common than expected.

An important caveat of molecular paternity studies is the common failure to sample all potential breeders, which may lead to incorrect estimates of variance in male reproductive success if a few highly successful males are not sampled. We were able to address this issue by sampling female-pup pairs which allowed us to extrapolate the paternal contribution to the genotype of each pup when the father had not been sampled. The low levels of relatedness between inferred paternal genotypes supported the observed low level of male reproductive skew and excluded the possibility that we had failed to sample a few highly successful males. In addition, patterns of male reproductive skew were consistent in the two rookeries studied, which have genetically distinct populations [44] and are located in opposite ends of the Gulf. Therefore, the observed weak levels of polygyny are likely widespread among sea lions in the Gulf, and not an artifact of sampling errors or of unique features in the sampled rookeries.

In pinnipeds, territorial behavior evolved as a way for males to monopolize groups of sexually receptive females thereby improving the reproductive success of territorial males [9], [11], [12]. The ability of males to monopolize females was not well supported by our paternity assignment results, as only a small fraction of female-pup pairs were assigned fathers that held territories at our study sites. This suggests that although female presence determines territory locations within a rookery [14], holding a territory does not guarantee matings with nearby females. Therefore, the direct benefits of male territoriality are unclear. Some authors have suggested territories could play a symbolic role akin to male displays in a lek, in that females are free to select mates from territorial males throughout a rookery [19], [21]. The pattern of paternity assignment at San Jorge provides some support for a lek-like mating system. Specifically, females tend to mate with territorial males near their pupping site and the number of males holding territories at the rookery is sufficient to account for the number of pups born each year. Contrary to our results, a symbolic role of territoriality should still result in high variation in male reproductive success, as only the most fit males (as determined by females) will secure the majority of matings [24]. This lek-like mating system has been documented in an Antarctic fur seal rookery where sexually receptive females move between male territories and seem to exert choice for males with high multi locus heterozygosity [45]; genetic paternity assignment at this rookery showed large variation in male reproductive success as expected in a lek-like mating system [39]. A recent study at one South American sea lion colony (*Otaria byronia*) also found strong support for a lek-like mating system, here females were observed briefly leaving their pupping site to solicit matings from nearby territorial males [46]. In this study, behavioral observations indicated high polygyny with 14% of the territorial males participating in 50% of all copulations [46]. In contrast to these studies, mating success did not vary significantly among territory holders at our study sites. This is confounded by the existence of variation in territorial behavior, where males more actively defend territories in which more females are present [30], which suggests that there is variation in territory quality (or the potential reproductive benefit to the territory holder) which can be measured by female presence.

Given the possibility of alternative mating tactics, the presence of breeding, non-territorial males could reduce male reproductive skew while maintaining an evolutionary advantage of territoriality. In this scenario, we expect that some males will maximize their fitness by defending territories whereas others (possibly smaller, less aggressive males) likely perform poorly as territory holders and will have a better chance of reproductive success by adopting an alternative mating tactic. Although some individuals will be more successful as non-territorial males than they would as territory holders, average reproductive success will always be highest for territorial males [47], [48]. If breeding, non-territorial males are all equally likely to sire offspring, in scenarios where these males greatly outnumber territorial males we would expect a reduction in overall male reproductive skew. For instance, if non-territorial males were able to secure 80% of the matings (this is the most extreme scenario justified by our data), even a large reproductive skew between territorial males for the remaining 20% of matings would be diluted when considering all matings.

The possibility that non-territorial males may regularly contribute to reproduction is supported by the fact that, on Los Islotes, females were less likely to sire pups with males which held territories within the study sites than expected at random (Table 3) and by the paucity of observed copulations at both rookeries. Seven-hundred and twenty observation hours at each site across four breeding seasons resulted in an average of only one copulation every two years per site. It is possible that copulations occur more frequently at night as a mechanism to prevent overheating in high daytime temperatures. A cumulative 20 hours of nighttime observations conducted with night vision goggles on San Jorge throughout July of 2008 resulted in no observed copulations. Thus, although nighttime observations were limited, it seems unlikely that copulation rate dramatically increases at night. The lack of observations suggests copulations may be occurring outside the rookeries' boundaries, such that some males intercept females traveling to and from the rookery on foraging trips. Strong evidence for a similar offshore, nonpolygynous mating tactic has been documented in the southern elephant seal (*Mirounga leonina*), a species that was previously thought to only mate at terrestrial breeding sites dominated by a few highly territorial males [49]. The alternative of sneaker males entering the sites and territories to mate is unlikely given that copulations were rarely observed (those that were observed always involved a territorial male), and the high level of vigilance exhibited by territorial males. A similar lack of observed copulations and a large proportion of pups not assigned a father was also proposed as suggestive of alternate mating tactics in the Galápagos sea lion [7].

As shown here, genetic analyses of paternity are critical to reveal true mating dynamics, as behavioral patterns may be misleading. For California sea lions, the maintenance of male territorial displays and behavioral observations were taken as indication of a lek-like mating system, where females show preference for certain males based on territorial displays or territory location and few males sire the majority of the pups [19]. However, our paternity analyses revealed a level of variation in male reproductive success much lower than expected in a lek-like mating system [24]. Instead, California sea lions in the Gulf appear to exhibit very weak polygyny, in which the function of territorial defense and the potential existence of alternative mating strategies remain to be clarified.
