# Correction: Weak Polygyny in California Sea Lions and the Potential for Alternative Mating Tactics

**DOI:** 10.1371/annotation/03fdf5e5-b986-49c7-aa43-202bdedd3a36

**Published:** 2012-04-26

**Authors:** Ramona Flatz, Manuela González-Suárez, Julie K. Young, Claudia J. Hernández-Camacho, Aaron J. Immel, Leah R. Gerber

There was an error in the funding statement. The correct funding statement is: The research was supported by the National Science Foundation (Grant no. 0347960 to LRG). MGS was funded in part by the European Community's Seventh Framework Programme (FP7/2007-2013; http://cordis.europa.eu/fp7) [^] under grant agreement n° 235897 and the Spanish Ministry of Science and Innovation (CGL2009-07301-BOS). The authors are grateful for a grant provided to CHC from Consejo Nacional de Ciencia y Tecnología (Scholarship No. 95802). The funders had no role in study design, data collection and analysis, decision to publish, or preparation of the manuscript. 

